# Assessment and selection of the best treatment alternative for infectious waste by modified Sustainability Assessment of Technologies methodology

**DOI:** 10.1186/s40201-016-0251-1

**Published:** 2016-05-27

**Authors:** Ata Rafiee, Kamyar Yaghmaeian, Mohammad Hoseini, Saeid Parmy, Amirhosein Mahvi, Masud Yunesian, Mehran Khaefi, Ramin Nabizadeh

**Affiliations:** Center for Solid Waste Research (CSWR), Institute for Environmental Research (IER), Tehran University of Medical Sciences, Tehran, Iran; Department of Environmental Health Engineering, School of Public Health, Tehran University of Medical Sciences, Tehran, Iran; Department of Environmental Health Engineering, School of Public Health, Shiraz University of Medical Sciences, Shiraz, Iran; Center for Air Pollution Research (CAPR), Institute for Environmental Research (IER), Tehran University of Medical Sciences, Tehran, Iran; Environmental and occupational health center, Ministry of health medical education, Tehran, Iran

**Keywords:** Health-care waste, Infectious waste treatment, SAT methodology, Hospital, Tehran

## Abstract

**Background:**

Improper treatment of infectious waste can cause numerous adverse environmental and health effects such as transmission of diseases through health personnel and other susceptible groups,who come in contact with such wastes. On the other hand, selection of appropriate treatment alternatives in infectious waste management has become a challenging task for public health authorities especially in developing countries. The objective of this paper is to select the best infectious waste treatment alternative by the modified Sustainability Assessment of Technologies (SAT) methodology, developed by the International Environmental Technology Center of the United Nations Environment Program (IETC-UNEP).

**Methods:**

SAT methodology consists of three main components, including screening, scoping and detailed assessment. In screening, different infectious waste treatment alternatives undergo screening using the finalized environmental and technical criteria. Short-listed treatment options from the previous step, then go through the comprehensive scoping and detailed assessment (2nd and 3rd components) which is more qualitative and quantitative in nature. An empirical case in Tehran, the largest city in Iran, is provided to illustrate the potential of the proposed methodology.

**Results:**

According to the final score, “Hydroclave”, was the most suitable infectious treatment technology. The ranking order of the treatment alternatives were “Autoclave with a shredder”, “Autoclave”, “Central Incineration” and “chemical treatment” on the basis of technical, economical, social and environmental aspects and their related criteria.

**Conclusions:**

According to the results it could be concluded that the top ranking technologies basically have higher scores in all the aspects. Hence it is easier to arrive at a decision for the final technology selection based on the principles of sustainability.

## Background

Today health-care wastes (HCWs) have become a substantial public health and environmental concern all over the world, particularly in developing countries [1, 2]. According to the World Health Organization (WHO), the term HCWs includes all the waste generated within health-care institutions, research centers and laboratories related to medical practices. HCWs can be classified into two major categories: “non-hazardous” or “general HCW_S_” which represents about 75–90 % of the total HCWs; and “hazardous” which represent 10–25 % of the total HCWs. Hazardous HCWs include, but are not limited to, infectious, chemical and radioactive wastes and may pose various environmental and health risks. Infectious waste refers to any waste type either known or suspected to contain pathogens (bacteria, viruses, parasites or fungi) in enough concentrations and/or quantities which lead to disease in susceptible hosts [3–5].

Although infectious waste is only a small part of the total HCWs, however, mismanagement in practices can cause this waste to be mixed with other non-hazardous waste [6, 7]. HCWs and infectious waste specifically may play an important role in the transmission and spread of many diseases such as human immunodeficiency virus (HIV), hepatitis B or C virus, and other agents associated with blood borne diseases [8–10]. A multi-language systematic review of HCW_S_ management in 40 low and middle-income countries worldwide declared that crucial problems in urban regions in Asia, Africa and the Middle East intensified by increasing quantities of HCWs and inappropriate treatment and disposal activities [11].

A survey conducted by WHO on HCWs management in 22 developing countries revealed that the proportion of health care facilities with improper waste treatment practices was between 18 and 64 % [12].

The goal of infectious waste treatment is to reduce the potential hazards of this type of wastes, and consequently protect public health and the environment [13]. To improve the HCWs management, implementation of appropriate methods is necessary [14–16]. However, selecting the best alternative for treating the HCWs and specially infectious waste is not always a simple task. To assess various HCWs management scenarios, Liu et al. [1] developed a hybrid multi-criteria decision method (MCDM) model by integrating the 2-tuple DEMATEL technique and fuzzy MULTIMOORA method for selecting the best HCWS treatment alternatives in Shanghai, China [1]. Liu et al. [16] introduced a MCDM model based on the fuzzy set theory and VIKOR method to identify the most suitable HCWs treatment alternative [16]. Dursun et al. [17] suggested two fuzzy MCDM techniques for assessing HCW_S_ treatment alternatives, which allow conducting an analysis based on a multi-level hierarchical structure and to incorporate uncertain data defined as linguistic variables into the analysis [17]. Karagiannidis et al. [18] assessed the thermal treatment processes of infectious wastes in Central Macedonia, Greece by analytic hierarchy process (AHP) [18]. Hsu et al. [19] used (AHP) method to objectively select medical waste disposal alternatives based on the results of interviews with experts in the field.

During the selection of treatment alternatives for HCWs, decision makers usually consider different criteria and sub-criteria for optimal decisions [19]. All the studies discussed above developed and applied different methods for selection of HCWs treatment alternatives. The main limitation of the existing decision analysis methods is that most of them are only mathematical models without focusing on important issues such as the sustainability concept. In response to the need for a technology assessment framework to identify and select the best possible environmental technology option, the International Environmental Technology Center of the United Nations Environment Program (IETC-UNEP) developed a new methodology known as Sustainable Assessment of Technologies (SAT). The focus of this methodology is both on the process and outcome, with an interest towards informed and participatory decision-making. The methodology employs a progressive assessment involving initial screening, scoping and detailed assessment. Importantly, the methodology takes a systems approach and stresses information, expertise and stakeholder participation [13]. In applying this methodology to developing countries, it seems necessary to make some changes on its criteria, based on local conditions. The aim of this study was to select the best treatment alternative for the infectious waste by modified Sustainability Assessment of Technologies (SAT) methodology.

## Methods

### Study area

The present study was performed in Tehran, the capital of Iran and one of the most crowded areas in Iran and in the Middle East, with eleven million inhabitants, in mid 2014 [20]. The average health-care waste generation in Tehran public hospitals is 65000 kg/day [6]. To illustrate the application of the suggested methodology for selecting the best alternative for infectious waste treatment, a case study conducted at the Imam Khomeini hospital complex. The complex, which is the largest and most advanced educational and medical center in Iran, is located in the center of Tehran in an area of 25 Hectares and a capacity of 1400 hospital beds out of which 1200 are considered active beds. The complex includes Imam Khomeini and Vali-e-Asr hospitals, cancer institute, radiology center, polyclinics and an emergency ward.

### Application of SAT methodology

As stated above, SAT is a suitable methodology for integrating technical, environmental, social, and economic considerations with main focus on environmental issues and developmental aspects. This methodology consists of three main steps, including screening, scoping and detailed assessment. In order to adapt the methodology to national conditions, country specific parameters and constraints, we made some changes on its criteria and applied modified methodology to select the best alternative.

#### Screening

In this step, at first baseline data, including information on HCWs generation rate, number of beds, average occupancies and identifying stakeholders were collected from the studied hospital. Then, common thermal, chemical and radiation infectious waste treatment alternatives were screened by using screening criteria based on objective yes/no type answers. The total of participants in screening step were 25 individuals, that included doctors, nurses, environmental health experts, and hospital manager, 5, 5, 14, and 1 respectively. The technologies that didn’t meet the basic criteria were directly excluded and the rest, were selected for further assessments. Autoclave, autoclave with a shredder, chemical treatment, hydroclave, demolizer, microwave, chem-Clav and central incineration were selected in this step. The criteria for this step described in SAT methodology included compliance with local environmental laws, compliance with national environmental laws, compliance with multilateral environmental agreements and consistency with WHO policies. Since Iran lacks local environmental laws, the criteria of “compliance with local environmental laws” weren’t considered in this study. Instead, in order to increase the accuracy of the results of this step, some specific scoping criteria which were applicable to this step were added. Table 1 show the modified screening criteria in the screening component.

**Table 1:** Adjusted Screening Step Worksheet (UNEP, 2012)

Criteria	Autoclave	Autoclave with aShredder	Hydroclave	Chem-Clav	Microwave	Chemical treatment	Demolizer	Central incineration
Compliance with national environmental laws	Y/N	Y/N	Y/N	Y/N	Y/N	Y/N	Y/N	Y/N
Compliance with multilateral environmental agreements	Y/N	Y/N	Y/N	Y/N	Y/N	Y/N	Y/N	Y/N
Consistency with WHO policies	Y/N	Y/N	Y/N	Y/N	Y/N	Y/N	Y/N	Y/N
Added criteria to screening step of the basic SAT methodology								
Meets capacity requirement	Y/N	Y/N	Y/N	Y/N	Y/N	Y/N	Y/N	Y/N
Availability of spare parts	Y/N	Y/N	Y/N	Y/N	Y/N	Y/N	Y/N	Y/N
Safe to use	Y/N	Y/N	Y/N	Y/N	Y/N	Y/N	Y/N	Y/N
Volume reduction	Y/N	Y/N	Y/N	Y/N	Y/N	Y/N	Y/N	Y/N
Mass reduction	Y/N	Y/N	Y/N	Y/N	Y/N	Y/N	Y/N	Y/N
Air emissions	Y/N	Y/N	Y/N	Y/N	Y/N	Y/N	Y/N	Y/N
Technology Economically Viable	Y/N	Y/N	Y/N	Y/N	Y/N	Y/N	Y/N	Y/N

*Y* yes, *N* no

#### Scoping

**Fig. 1:**
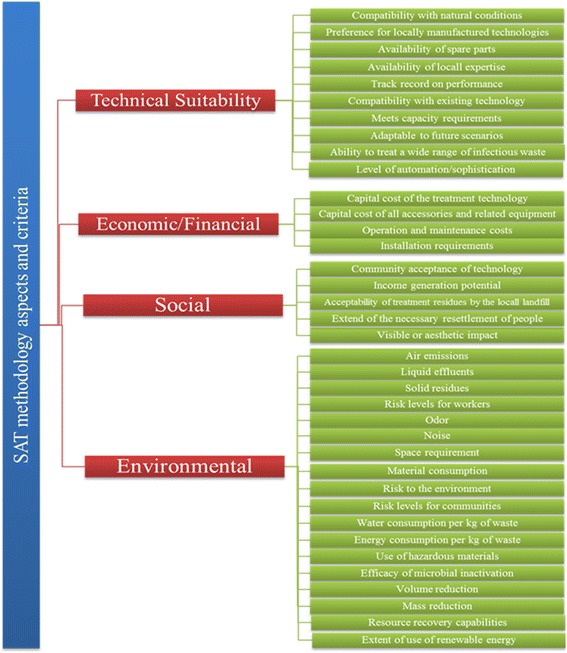
SAT methodology aspects and their related criteria

After the screening step, technologies that did not qualify for the conditions were excluded and other technologies were assessed against specific criteria. Demolizer, Microwave and Chem-Clav technologies were eliminated in the earlier step and the shortlisted treatment alternatives (including autoclave, autoclave with a shredder, chemical treatment, central incineration and hydroclave) then underwent the comprehensive scoping assessment. The scoping step, which is a comprehensive and qualitative type (High/Medium/Low) assessment, uses selected technical, economic, social and environmental criteria (Fig. 1). This step lends an advantage in narrowing the decision range of scores, for a particular criteria in the detailed assessment level. For instance, if low/medium/Highscores are assigned on a basis of a scale of 0–9, then evaluation as ‘medium’ would scope the scores say between 4 and 6. This allows a narrowing of the range and therefore better compliance of opinions and thus reduced subjectivity. In order to select the most preferred infectious waste treatment technology, different groups of experts in HCWs field, were asked to fill the scoping questionnaire. The experts were divided into two groups. Group A, included 25 academic members of the Environmental science and Environmental Health Engineering departments across the country (associate and full professors), 5 experts from infectious waste disposal companies and 5 experts from the Ministry of Health and Medical Education. Group B included 35 graduate students (20 PhD and 15 masters students) in environmental health engineering. At first, the experts were requested to rank the relative importance of each four topics (technical suitability, economic/financial, social/cultural and environmental) by scoring from 0 to 100 so that the sum of all the ranking scores becomes 100. Each four topics were ranked environmental (45), economic/financial (25), technical suitability (20), and social/cultural (10). Then, experts asked to establish weighting factors (0 to 9) for each criteria (1 to 3 for low, 4 to 6 for medium, 7 to 9 for high). Besides, in order to determine the most important criteria in each aspects, the experts were asked to score the each criterion. The importance of each criteria assigned by the experts divided into five groups: very low (1), low (2), medium (3), high (4), essential (5).

#### Detailed assessment

The technologies with best overall ratings from the scoping step were selected for further assessment in this step. Different multi-criteria decision methods (MCDMs) can be used for quantitative assessment of treatment alternatives in this step. In this study and in order to keep the integrity of the methodology, a weighted sum matrix method was used. The weight assigned to each criterion within a category was based on the importance given by the expert’s judgment. The number or rating assigned to each technology based on the expert reflects how well the technology complies with each defined criteria. It should be noted that there were 10 technical, 4 financial, 5 social and 18 environmental criteria. For each criterion multiplying factors need to be calculated. For this at the first step, maximum scores for each topic were calculated as follows [13]:

Maximum score for Technical Suitability (MST):

$$ \mathrm{M}\mathrm{S}\mathrm{T} = 9 \times \left({\mathrm{W}}_1+{\mathrm{W}}_2+{\mathrm{W}}_3+{\mathrm{W}}_4+{\mathrm{W}}_5+{\mathrm{W}}_6+{\mathrm{W}}_7+{\mathrm{W}}_8+{\mathrm{W}}_9+{\mathrm{W}}_{10}\right) $$

Maximum score for Economic/Financial (MSEn):

$$ \mathrm{MSEc} = 9 \times \left({\mathrm{W}}_{11}+{\mathrm{W}}_{12}+{\mathrm{W}}_{13}+{\mathrm{W}}_{14}\right) $$

Maximum score for Social/Cultural (MSS):

$$ \mathrm{M}\mathrm{S}\mathrm{S} = 9 \times \left({\mathrm{W}}_{15}+{\mathrm{W}}_{16}+{\mathrm{W}}_{17}+{\mathrm{W}}_{18}+{\mathrm{W}}_{19}\right) $$

Maximum score for Environment (MSEn):

$$ \begin{array}{l}\mathrm{MSEn} = 9 \times \Big({\mathrm{W}}_{20}+{\mathrm{W}}_{21}+{\mathrm{W}}_{22}+{\mathrm{W}}_{23}+{\mathrm{W}}_{24}+{\mathrm{W}}_{25}+{\mathrm{W}}_{26}+{\mathrm{W}}_{27}+{\mathrm{W}}_{28}+\hfill \\ {}+{\mathrm{W}}_{29}+{\mathrm{W}}_{30}+{\mathrm{W}}_{31}+{\mathrm{W}}_{32}+{\mathrm{W}}_{33}+{\mathrm{W}}_{34}+{\mathrm{W}}_{35}+{\mathrm{W}}_{36}+{\mathrm{W}}_{37}\Big)\hfill \end{array} $$

Where Wi is the weight of each criterion.

Then, the multiplying factors (MF) for each topic were calculated as follows:

Multiplying factors for Technical Suitability aspect:

$$ \begin{array}{l}\begin{array}{l}\mathrm{M}{\mathrm{F}}_1 = {\mathrm{W}}_1 \times {\mathrm{R}}_{\mathrm{T}}\kern0.1em /\kern0.1em \mathrm{M}\mathrm{S}\mathrm{T}\\ {}.\\ {}.\\ {}.\end{array}\hfill \\ {}\mathrm{M}{\mathrm{F}}_{10}={\mathrm{W}}_{10} \times {\mathrm{R}}_{\mathrm{T}}\kern0.1em /\kern0.1em \mathrm{M}\mathrm{S}\mathrm{T}\hfill \end{array} $$

Multiplying factors for Economic/Financial aspect:

$$ \begin{array}{l}\mathrm{M}{\mathrm{F}}_{11} = {\mathrm{W}}_{11} \times {\mathrm{R}}_{\mathrm{Ec}}\kern0.1em /\kern0.1em \mathrm{M}\mathrm{SEc}\ \mathrm{M}{\mathrm{F}}_{13} = {\mathrm{W}}_{13} \times {\mathrm{R}}_{\mathrm{Ec}}\kern0.1em /\kern0.1em \mathrm{M}\mathrm{SEc}\hfill \\ {}\mathrm{M}{\mathrm{F}}_{12} = {\mathrm{W}}_{12} \times {\mathrm{R}}_{\mathrm{Ec}}\kern0.1em /\kern0.1em \mathrm{M}\mathrm{SEc}\ \mathrm{M}{\mathrm{F}}_{14} = {\mathrm{W}}_{14}\times {\mathrm{R}}_{\mathrm{Ec}}\kern0.1em /\kern0.1em \mathrm{M}\mathrm{SEc}\hfill \end{array} $$

Multiplying factors for Social/Cultural aspect:

$$ \begin{array}{l}\mathrm{M}{\mathrm{F}}_{15} = {\mathrm{W}}_{15}\times {\mathrm{R}}_{\mathrm{S}}\kern0.1em /\kern0.1em \mathrm{M}\mathrm{S}\mathrm{S}\ \mathrm{M}{\mathrm{F}}_{18} = {\mathrm{W}}_{18}\times {\mathrm{R}}_{\mathrm{S}}\kern0.1em /\kern0.1em \mathrm{M}\mathrm{S}\mathrm{S}\hfill \\ {}\mathrm{M}{\mathrm{F}}_{16} = {\mathrm{W}}_{16}\times {\mathrm{R}}_{\mathrm{S}}\kern0.1em /\kern0.1em \mathrm{M}\mathrm{S}\mathrm{S}\ \mathrm{M}{\mathrm{F}}_{19} = {\mathrm{W}}_{19}\times {\mathrm{R}}_{\mathrm{S}}\kern0.1em /\kern0.1em \mathrm{M}\mathrm{S}\mathrm{S}\hfill \\ {}\mathrm{M}{\mathrm{F}}_{17} = {\mathrm{W}}_{17}\times {\mathrm{R}}_{\mathrm{S}}\kern0.1em /\kern0.1em \mathrm{M}\mathrm{S}\mathrm{S}\hfill \end{array} $$

Multiplying factors for Environmental aspect:

$$ \begin{array}{l}\mathrm{M}{\mathrm{F}}_{20} = {\mathrm{W}}_{20} \times {\mathrm{R}}_{\mathrm{T}}\kern0.1em /\kern0.1em \mathrm{M}\mathrm{S}\mathrm{T}\hfill \\ {}.\hfill \\ {}.\hfill \\ {}.\hfill \\ {}\mathrm{M}{\mathrm{F}}_{37}={\mathrm{W}}_{37} \times {\mathrm{R}}_{\mathrm{T}}\kern0.1em /\kern0.1em \mathrm{M}\mathrm{S}\mathrm{T}\hfill \end{array} $$

## Results and discussion

### Quantities and characteristics of wastes generated in studied hospital complex

The average HCWs generated by Imam Khomeini hospital complex during March to June 2014 was 3270 kilograms per day (Table 2). The percentage of general, infectious and sharps waste were 47.1, 42.2 and 10.7 %, respectively. WHO reported that 75- 90 % of total HCWs are general waste and 10- 25 % are infectious and hazardous wastes [3]. Results showed that the infectious and hazardous wastes measured in this study were higher than those reported by WHO. This indicates that segregation of different types of wastes wasn’t properly implemented in the studied hospitals. According to the results, the mean generation rate for total, general, infectious and sharp wastes were 2.72,1.28, 1.15, and 0.29 kg occupied bed^-1^ day^-1^,respectively. HCWs generation rate previously reported in different studies in Iran which conducted in other cities including Mashhad, Tabriz, Isfahan, and Shiraz have been reported in the range of 2.6 to 4.45 kg occupied bed^-1^ day^-1^ for total HCWs,1.5 to 2.44 kg occupied bed^-1^ day^-1^ of general for non-infectious waste, and 1.039 to 1.59 kg occupied bed^-1^ day^-1^ for infectious waste [21–24]. Furthermore, Farzadkia et al 2008, reported a mean medical waste generation rate of 2.75 kg occupied bed^-1^ day^-1^ which was similar to the results of this study [25]. Among the different wards of the studied hospitals, operation rooms had the highest infectious waste generation rate. This can be caused by the types of services provided in this ward which comprises of various surgical procedures. A significant difference was observed based on weighed infectious wastes, which showed a statistically higher (*p* < 0.001) quantity of HCWs in summer than that of winter.

**Table 2:** Imam Khomeini hospital complex average healthcare waste generation

Names of hospitals	Quantity of wastes (kg d^-1^)		
	General waste	Infectious waste	Sharps waste
Imam Khomeini	857.32	795.72	115.15
Vali-Asr	418.68	349.07	116.95
Cancer Institute	264	235.21	117.9
Total	1540	1380	350

### Screening step

After gathering the stakeholders comments and choices, treatment alternatives that didn’t meet the basic criteria, were excluded. The results of the screening step are presented in Table 3. As shown in Table 3, demolizer, Chem-cloth and microwave were excluded at this step, which may be due to the inability of these technologies to reduce the volume and mass of infectious waste, inadequate capacity of them or lack of stakeholders familiarity with these technologies.

**Table 3:** Screening step results

Technology	Positive score	Negative score	Net score
Hydroclave	75	24	51
Autoclave + Shreeder	67	31	36
Central incineration	63	32	31
Autoclave	55	33	22
	Excluded alternatives		
Chemical treatment	51	36	15
Microwave	34	43	-9
Demolizer	23	45	-15

### Scoping step

Qualitative assessment based on the expert’s opinion was performed during this step. The results of the scoping step for each aspect provided in Tables 4 and 5.

**Table 4:** Qualitative assessment of Technical suitability, Economic/Financial and Social/Cultural aspects

Alternatives						
Criteria		Autoclave	Autoclave with a shredder	Chemical treatment	Central incineration	Hydroclave
Technical suitability	Compatibility with natural conditions	Medium	High	Medium	Medium	High
	Preference for locally manufactured technologies	Medium	Medium	Medium	Low	Medium
	Availability of spare parts	Medium	Medium	High	Medium	Medium
	Availability of local expertise	Medium	Medium	Medium	Medium	Medium
	Track record on performance	Medium	High	Medium	High	High
	Compatibility with existing technology	Medium	Medium	Medium	Medium	High
	Meets capacity requirement	Medium	Medium	Medium	High	High
	Adaptable to future situations	Medium	Medium	Medium	High	High
	Ability to treat a wide range of infectious wastes	Medium	High	Medium	High	High
	Level of automation/sophistication	Medium	Medium	High	Medium	Medium
Economic/Financial	Capital cost of the treatment Technology	High	Medium	High	Low	Medium
	Capital costs of all accessories and related equipment	Medium	Medium	High	Low	Medium
	Operation and maintenance costs	Medium	Medium	Medium	Low	Medium
	Installation requirements	Medium	Medium	High	Medium	Medium
Social/Cultural	Community acceptance of the technology	High	High	Medium	Low	High
	Income generation potential	Low	Low	Low	High	Low
	Acceptability of treatment residues by the local landfill	Medium	Medium	Medium	Medium	Medium
	Extent of necessary resettlement of people	High	High	Medium	Low	High
	Visible or aesthetic impact	Medium	Medium	Medium	Low	High

**Table 5:** Qualitative assessment of **Environmental** aspect

Alternatives					
Criteria	Autoclave	Autoclave with a shredder	Chemical Treatment	Central incineration	Hydroclave
Air emissions	Medium	Medium	Low	Low	Medium
Liquid effluents	Medium	Medium	Low	Medium	High
Solid residues	Medium	Medium	Low	High	Medium
Risk levels for workers	Medium	Medium	Low	Medium	Medium
Odor	Medium	Medium	Low	Medium	Medium
Noise	Medium	High	High	Medium	Medium
Space requirement	Medium	Medium	High	Low	Medium
Material consumption	Medium	High	Low	Medium	High
Risk to the environment	Medium	High	Low	Low	High
Risk levels for communities	Medium	High	Medium	Low	High
Water consumption per kg of waste	Low	Medium	Medium	High	High
Energy consumption per kg of waste	Medium	Medium	High	Medium	Medium
Use of hazardous materials	High	High	Low	High	High
Efficacy of microbial inactivation	Medium	High	Medium	High	High
Volume reduction	Low	Medium	Low	High	High
Mass reduction	Low	Low	Low	High	High
Resource recovery capabilities	Medium	Medium	Low	Low	Medium
Extent of use of renewable energy	Medium	Medium	Low	Medium	High

As the results show, autoclave obtained a medium score for all technical suitability aspects criteria. A similar result was observed for autoclave with a shredder, chemical treatment and central incineration. Also for most technical suitability criteria, the hydroclve obtained high scores.

Regarding the economic/financial aspect criteria, as shown in Table 4, high and low scores were obtained for chemical treatment and central incineration, respectively. Regarding this aspect, chemical treatment was the preferable choices of experts. Moreover, hydroclave and autoclave with a shredder obtained the medium scores for all economical aspects criteria. The similar result was observed for autoclave.

As shown in Table 4, regarding the social aspect, the high, medium and low scores for almost criteria were obtained for hydroclave, chemical treatment, and central incineration, respectively. Also, results were similar for autoclave and autoclave with a shredder regarding social aspect.

As shown in Table 5, based on qualitative assessment, hydroclave and chemical treatment obtained high and low scores for almost environmental aspect criteria, respectively. Autoclave, autoclave with a shredder and central incineration obtained medium scores for the most environmental aspect criteria.

### Detailed assessment

**Fig. 2:**
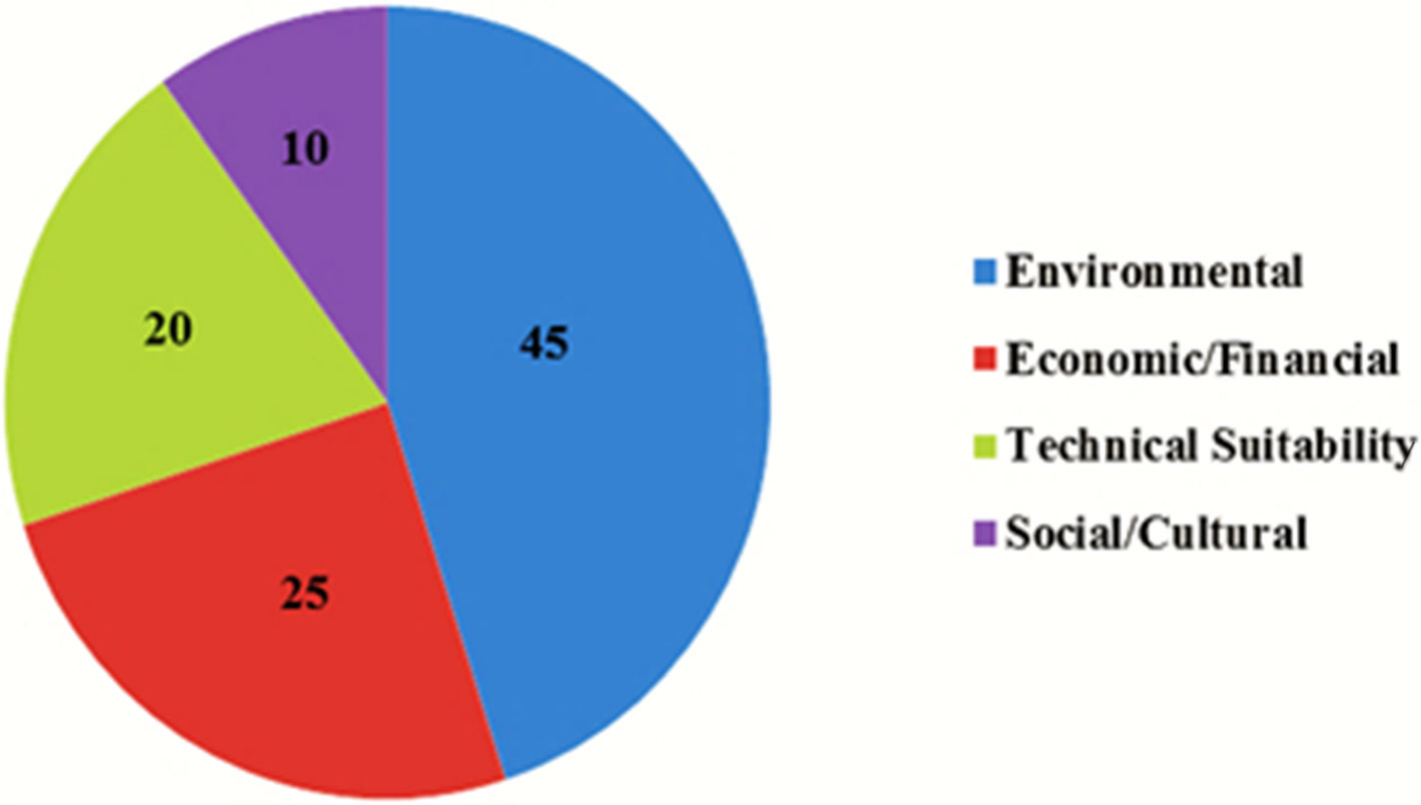
The distribution of scores for technical,economic, social and environmental aspects

**Fig. 3:**
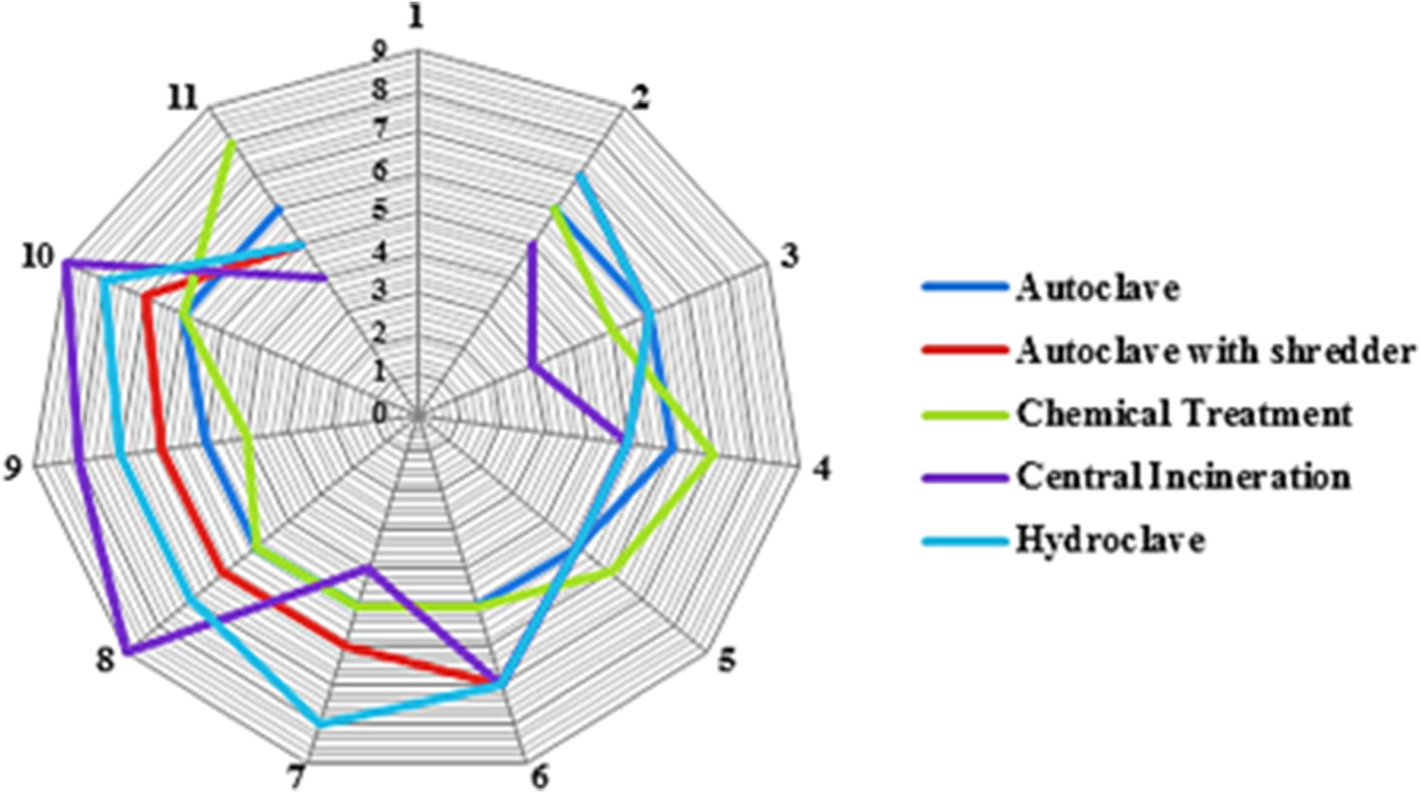
Star diagram for detailed assessment: Technical suitability

**Fig. 4:**
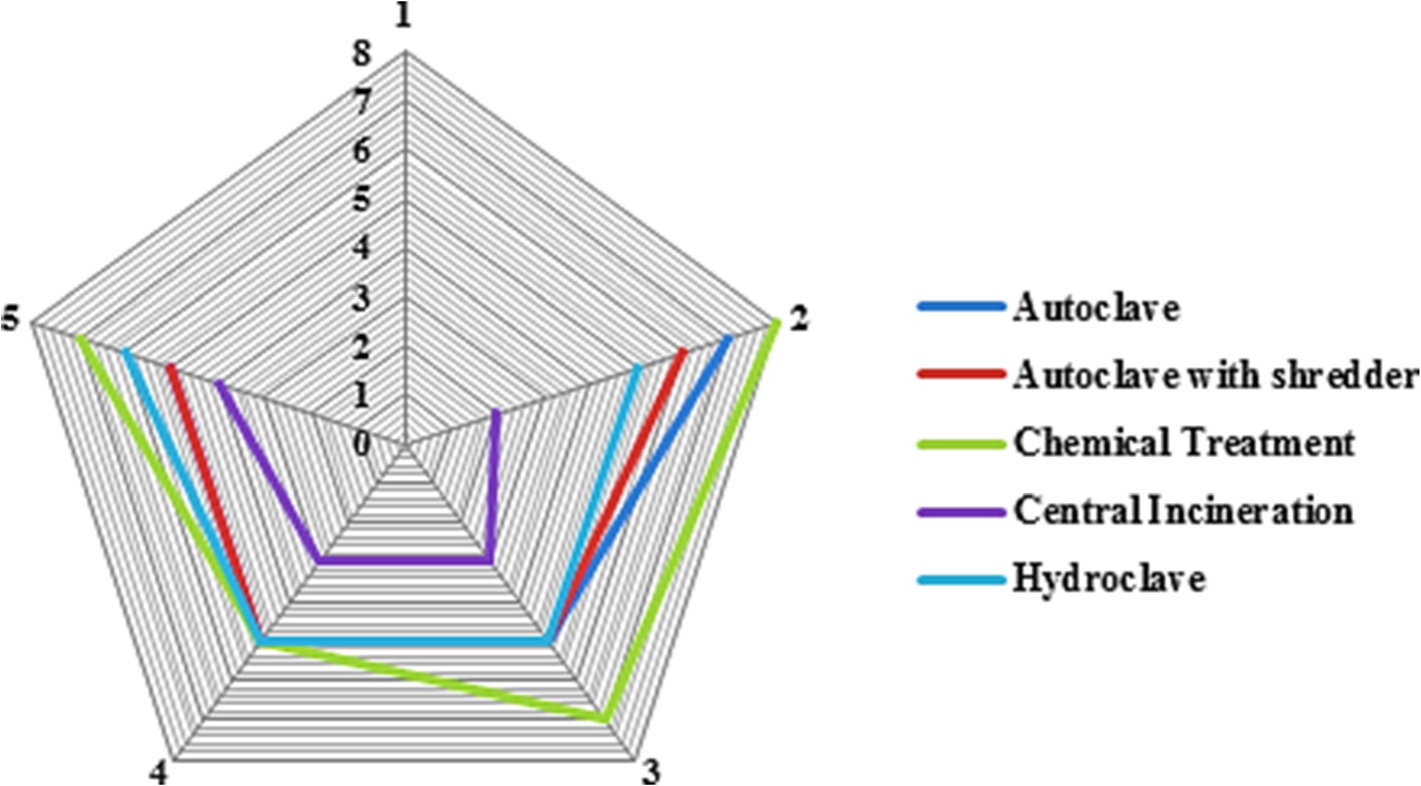
Star diagram for detailed assessment: **Economic/Financial**

**Fig. 5:**
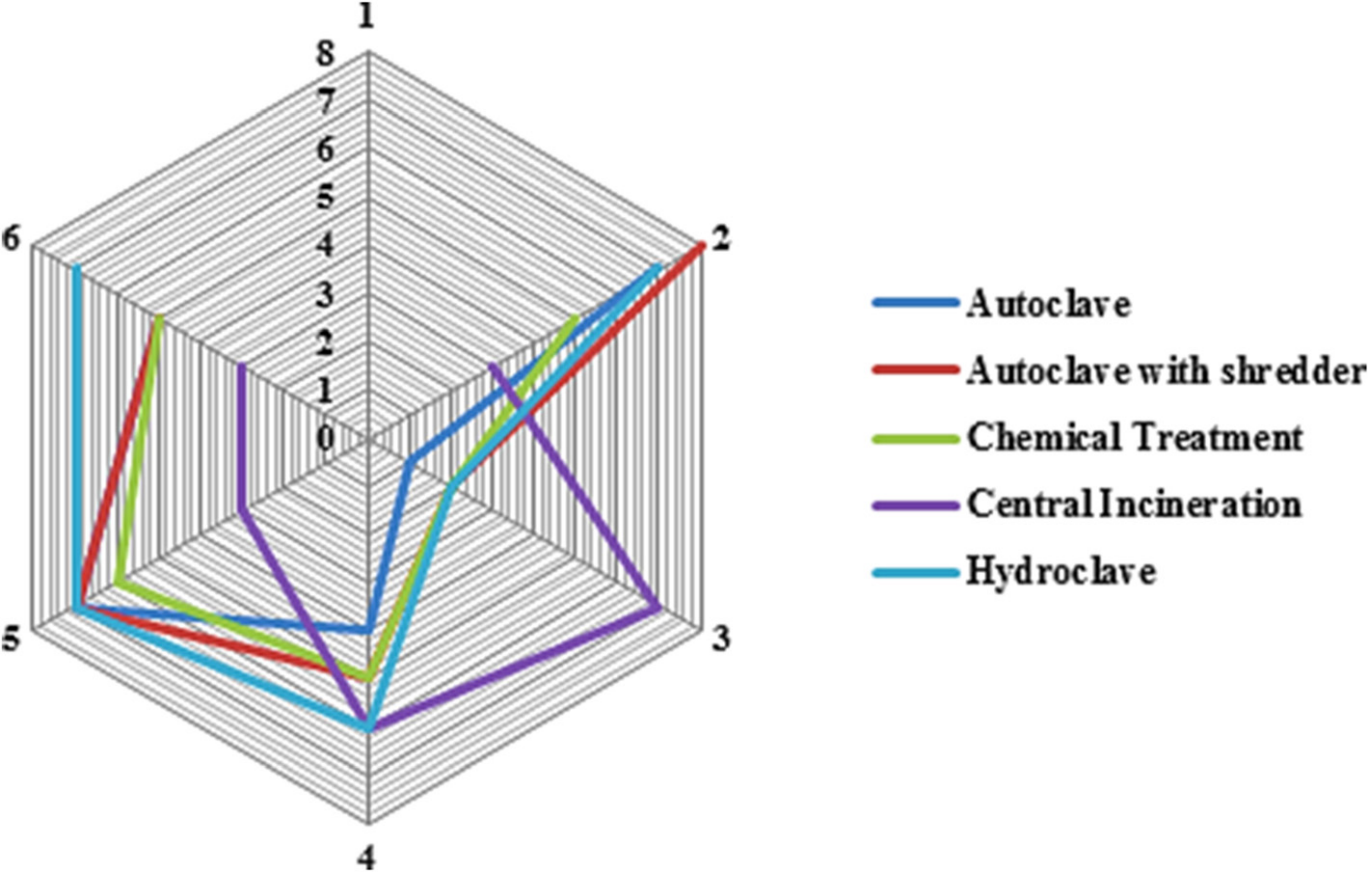
Star diagram for detailed assessment: **Social/Cultural**

**Fig. 6:**
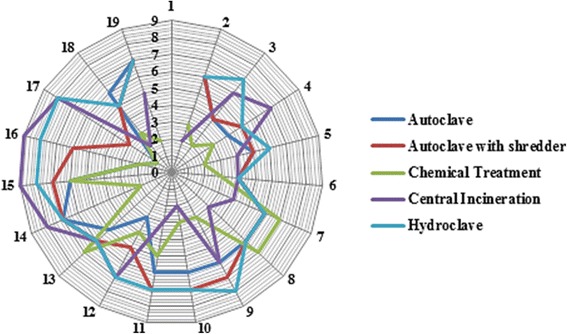
Star diagram for detailed assessment: **Environmental**

**Fig. 7:**
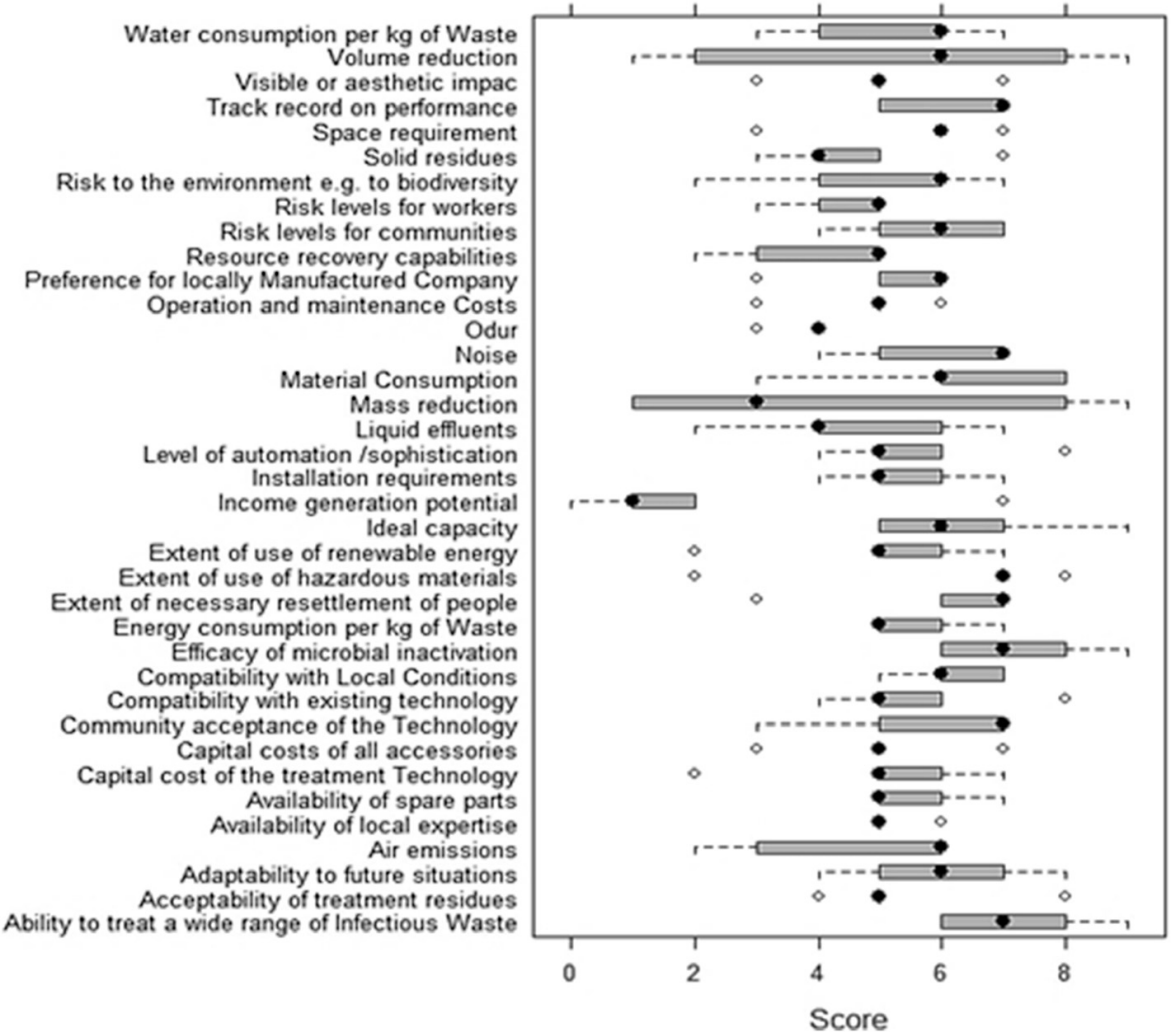
The box and whisker of scores for each criteria acquired by treatment alternatives

Different MCDMs can be used to perform a detailed assessment step. In this study, to maintain the integrity of the methodology, the weighted sum matrix method was used. The results of this step for each aspect are presented in Tables 6 and 7. The distribution of technical, economic, social and environmental aspects considered by the experts is shown in Fig. 2. The final scores obtained by different treatment alternatives for the technical suitability, economical, social and environmental aspects related criteria, are shown as a radar diagram provided in Figs. 3, 4, 5 and 6. Also, the distribution of the final weights of criteria for alternatives is presented in Fig. 7.

**Table 6:** Scores obtained for different Technical Suitability, Economic/Financial and Social/Cultural criteria

Alternatives											
Criteria		Autoclave		Autoclave with a shredder		Chemical Treatment		Central incineration		Hydroclave	
		Score	Score × MF	Score	Score × MF	Score	Score × MF	Score	Score × MF	Score	Score × MF
Technical suitability	Compatibility with natural conditions	6	1.45	7	1.81	6	1.33	5	0.93	7	1.68
	Preference for locally manufactured technologies	6	1.45	6	1.33	5	0.93	3	0.33	6	1.23
	Availability of spare parts	6	1.45	5	0.93	7	1.81	5	0.93	5	0.85
	Availability of local expertise	5	1.01	5	0.93	6	1.33	5	0.93	5	0.85
	Track record on performance	5	1.01	7	1.81	5	0.93	7	1.81	7	1.68
	Compatibility with existing technology	5	1.01	6	1.33	5	0.93	4	0.59	8	2.19
	Meets capacity requirement	5	1.01	6	1.33	5	0.93	9	3.00	7	1.68
	Adaptable to future situations	5	1.01	6	1.33	4	0.59	8	2.37	7	1.68
	Ability to treat a wide range of infectious wastes	6	1.45	7	1.81	6	1.33	9	3.00	8	2.19
	Level of automation/sophistication	6	1.45	5	0.93	8	2.37	4	0.59	5	0.85
Economic/Finanial	Capital cost of the treatment technology	7	6.19	6	4.75	8	8.47	2	0.53	5	3.31
	Capital costs of all accessories and related equipment	5	3.16	5	3.31	7	6.48	3	1.19	5	3.31
	Operation and maintenance costs	5	3.16	5	3.31	5	3.31	3	1.19	5	3.31
	Installation requirements	5	3.16	5	3.31	7	2.02	4	2.12	6	4.75
Social/Cultural	Community acceptance of the technology	7	2.27	8	2.63	5	1.03	3	0.37	7	2.02
	Income generation potential	1	0.05	2	0.16	2	0.16	7	2.02	2	0.16
	Acceptability of treatment residues by the local landfill	4	0.74	5	1.03	5	1.03	6	1.48	6	1.48
	Extent of necessary resettlement of people	7	2.27	7	2.02	6	1.48	3	0.37	7	2.02
	Visible or aesthetic impact	5	1.16	5	1.03	5	1.03	3	0.37	7	2.02

**Table 7:** Scores obtained for different Environmental criteria

Criteria	Alternatives									
	Autoclave		Autoclave with a shredder		Chemical Treatment		Central incineration		Hydroclave	
	Score	Score × MF	Score	Score × MF	Score	Score × MF	Score	Score × MF	Score	Score × MF
Air emissions	6	2.07	6	1.75	3	0.44	2	0.20	6	1.75
Effluents	4	0.92	4	0.78	2	0.20	6	1.75	7	2.40
Solid residues	4	0.92	5	1.23	3	0.44	7	2.40	5	1.23
Risk levels for workers	5	1.44	5	1.23	2	0.20	4	0.78	6	1.75
Odor	4	0.92	4	0.78	3	0.44	4	0.78	4	0.78
Noise	6	2.07	6	1.8	7	2.40	4	0.78	6	1.75
Space requirement	6	2.07	6	1.75	7	2.40	3	0.44	6	1.75
Material consumption	6	2.07	7	2.40	3	0.44	6	1.75	8	3.14
Risk to the environment	6	2.07	7	2.40	3	0.44	2	0.20	7	2.40
Risk levels for communities	6	2.07	7	2.40	5	1.23	3	0.44	7	2.40
Water consumption per kg of waste	3	0.52	5	1.25	4	0.8	7	2.40	7	2.40
Energy consumption per kg of waste	5	1.44	6	1.75	7	2.40	6	1.75	6	1.75
Use of hazardous materials	7	2.82	7	2.40	2	0.20	8	3.2	7	2.40
Efficiencyof microbial inactivation	6	2.07	7	2.40	6	1.75	9	3.97	8	3.14
Volume reduction	2	0.23	6	1.75	2	0.20	9	3.97	8	3.14
Mass reduction	1	0.06	3	0.44	1	0.05	8	3.12	8	3.14
Resource recovery capabilities	6	2.04	5	1.23	3	0.44	2	0.20	5	1.23
Extent of use of renewable Energy	7	2.38	7	2.73	2	0.20	5	1.23	7	2.40
Final Score		28.18		30.51		14.68		29.39		39

As the results show, environmental aspect was the most important aspect of the SAT methodology based on the expert’s opinion. Among the different criteria in technical suitability aspect, the most important criteria considered by experts were ability to treat a wide range of infectious wastes, ideal capacity, track record on performance and availability of spare parts and usage of local materials, respectively. Regarding the economic/financial aspect, experts considered the capital cost of the treatment technology, operation and maintenance costs and installation requirements as the most important criteria, respectively. The most important criteria in the social aspect were community acceptance of the technology, acceptability of treatment residues by the local landfill and extent of necessary resettlement of people, respectively. In the environmental aspect, experts considered the criteria of efficiency of microbial inactivation, volume and mass reduction, emissions, risk levels for communities and the environment to be the most important criteria, respectively.

As the results show, regarding the technical suitability aspects, the highest scores were obtained for hydroclave and central incineration technologies. These results indicate that there is no significant difference between hydroclave and central incineration in terms of technical suitability (*p* < 0.001) that indicates the suitability of incineration for treatment of infectious waste as well as hydroclave. Moreover, among various technical suitability criteria, the highest scores were obtained for two criteria, including ideal capacity, and ability of treating a wide range of infectious waste. These high scores were obtained for incineration technology. These results are consistent with results reported by Pudussery who studied different medical waste management options in the Norfolk and Norwich university hospital and in which the incineration obtained the highest scores for type of waste treated, and volume and mass reduction of HCWs [26].

Regarding the economic/financial aspect, as shown in Table 6 and Fig. 4, the highest and lowest scores were obtained for chemical treatment and central incineration, respectively. The capital cost of implementation for chemical treatment is lower than those for other alternatives which is the main advantage of this treatment technology in terms of economic and financial aspect. On the other hand, although incineration acquired ahigh score regarding the technical suitability, however the higher capital and also operational and maintenance costs for incineration compared with other treatment alternatives are one of the disadvantages of this treatment technology regarding the financial aspect. These results are consistent with results reported by Pudussery, indicating that incinerators have higher capital and maintenance costs than other treatment alternatives [26].

As shown in Table 6, regarding the social aspect the highest score was obtained for hydroclave. The similar result was observed for autoclave and autoclave with a shredder. Among various criteria in social/cultural aspect, the highest score was obtained for the community acceptance of the technology which was acquired for autoclave with a shredder. On the other hand, the lowest score was obtained for the criterion of income generation potential.

One of the most important aspects of SAT methodology is the environmental aspect which has 18 specific criteria. According to the results that presented in Table 7 and Fig. 6, hydroclave and chemical treatment got highest and lowest scores for the environmental aspect, respectively. The highest score among environmental criteria is associated with central incineration for efficiency of microbial inactivation and volume reduction that are the main advantages of it. Also, the lowest scores in environmental criteria are associated with the autoclave and chemical treatment for mass reduction criteria.

As shown in Table 8, regarding all aspects of SAT methodology, the highest score was obtained for hydroclave. However, it may so happen that the selected best technology may be found to be inadequate or inappropriate in the future. This may happen due to changes in the situation, local requirements, legislations, or even the new developments in technology. Similar studies have been conducted in other countries. For instance, in a comparative study, Karagiannidis et al [18] assessed the thermal treatment processes of infectious hospital wastes in Central Macedonia, Greece via the analytic hierarchy process (AHP). The results demonstrated that a centralized hydroclave or autoclave plant is the best alternative, which was similar to our findings [18]. A study was conducted in china that proposed a hybrid multi-criteria decision making (MCDM) model by integrating the 2-tuple DEMATEL technique and fuzzy MULTIMOORA method for selection of HCWs treatment alternatives. Results showed that steam sterilization was found to be the most suitable HCWs treatment technology [1]. Puddussery utilized MCDA matrix method to find out the best technology for the on-site HCWs treatment in at the NORFOLK and NORWICH university hospital, in England. According the results, incineration was the optimum technology for the hospital [26]. Dursun et al proposed two fuzzy MCDM techniques which were based on fusion of fuzzy information, 2-tuple linguistic representation model, and TOPSIS for the evaluation of HCWs treatment alternatives for Istanbul. According to the results of the study, steam sterilization was determined as the most suitable treatment technology [17].

**Table 8:** Final score of infectious waste treatment alternatives

Alternatives					
Criteria	Autoclave	Autoclave with a shredder	Chemical Treatment	Central incineration	Hydroclave
Technical Suitability	12.3	13.54	12.48	14.48	14.88
Economical	15.67	14.69	20.28	5.03	14.69
Social	6.49	6.87	4.73	4.61	7.7
Environmental	28.18	30.51	14.68	29.39	39
Total	62.64	65.61	52.17	53.51	76.27

**Fig. 8:**
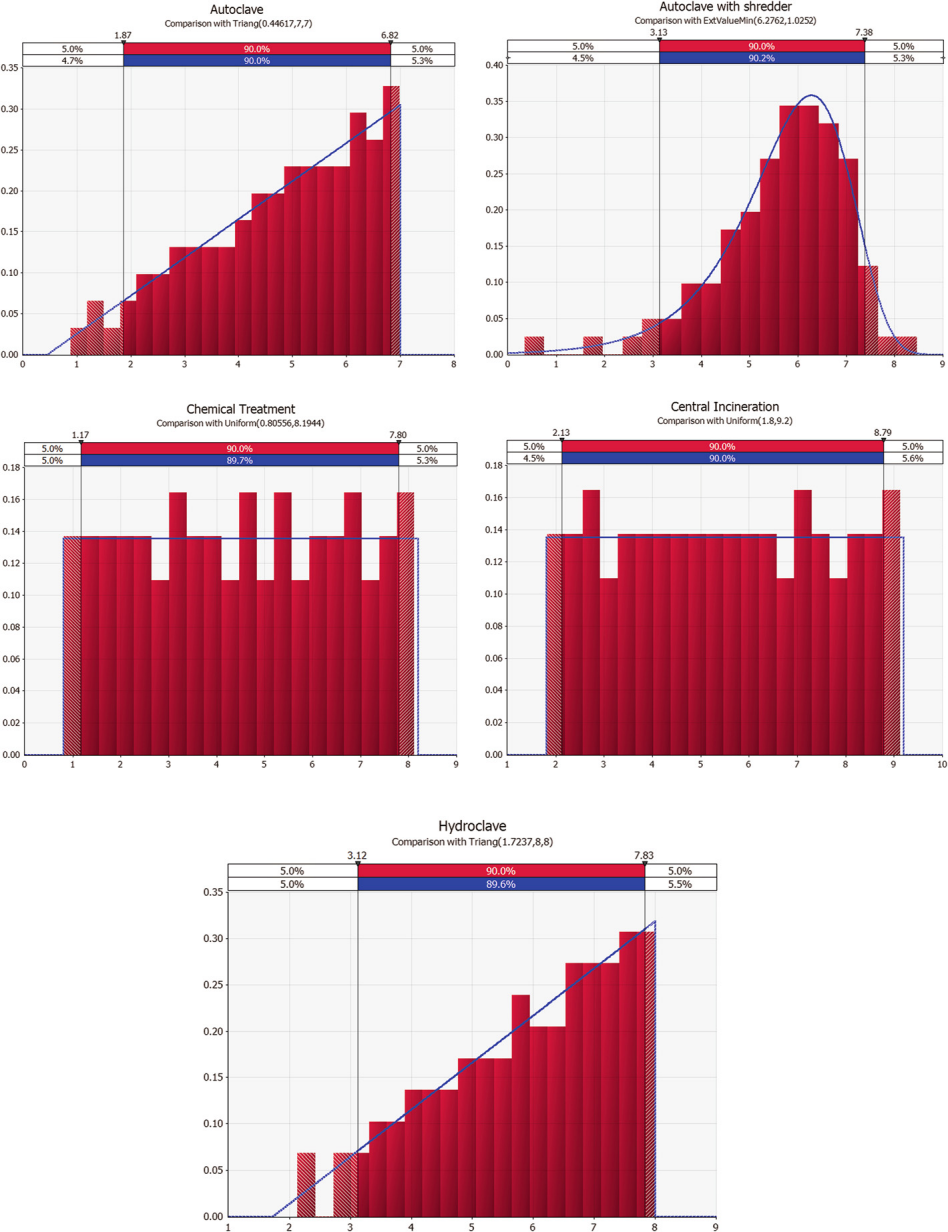
The distribution of final weights for treatment alternatives

The box and whisker of scores for each criterion, are shown in Fig. 7. As the results show, for volume reduction 50 % of scores, were between 2 to 8. For mass reduction 50 % of scores were between 1 to 8. This result indicated the wide range of scores for these criteria. In addition, the distribution of final weights for treatment alternatives is given in Fig. 8. For chemical treatment and central incineration, the type of the distribution was uniform. Distribution types for autoclave and hydroclave were the triangle type. For autoclave with a shredder, extreme value min distribution was observed.

## Conclusion

The most appropriate infectious waste treatment technology was selected based on the highest score. According to obtained final score, hydroclve, was the most suitable infectious waste treatment technology. The ranking order of the alternative treatments were Autoclave with a shredder, Autoclave, Central Incineration and Chemical treatment on the basis of the technical, economic, social and environmental aspects and their related criteria. So, as mentioned in SAT methodology, it could be concluded that the top ranking technologies basically have higher scores in all the aspects. Hence it is easier to arrive at a decision for the final technology selection based on the principles of sustainability. One limitation of the present study is that the results are subjected to the reliability on the response of experts on the questions in the survey. Also, the questionnaire surveys were time consuming and only 70 out of 150 questionnaires send were replied. Although to the best of our knowledge, this study is the first comprehensive study in which SAT methodology have been used to select the best treatment alternative for infectious waste in developing countries; however, there are a number of areas in which further research could be conducted. First, since the objective weights of the criteria were not considered and also the subjective weights were dependent on the experts’ personal judgments, which may result in some errors or mistakes, there is a need to develop a new approach accounting subjective and objective weights of criteria simultaneously. Second, the fuzzy MCDMs for SAT methodology can be performed based on the most important criteria in each aspect determined in this study. Finally, the present study was conducted in a developing country. So it would be useful to conduct similar studies in developed countries and comparing the results with the results of this study.

## Abbreviations

AHP: analytic hierarchy process
CSWR: Center for Solid Waste research
HCWs: health-care wastes
HIV: human immunodeficiency virus
IER: Institute for Environmental Research
IETC-UNEP: International Environmental Technology Center of the United Nations Environment Program
MCDM: multi-criteria decision method
SAT: Sustainable Assessment of Technologies methodology
WHO: World Health Organization
